# How to Use High Potencies

**Published:** 1882-02

**Authors:** 


### HOW TO USE HIGH POTENCIES.

In order to use these potencies successfully, the following conditions must be fulfilled:

1. Every case of disease must be investigated according to its most particular symptoms, and the greatest precision and circumspection must be used in the selection of the homœopathic agent. This selection must not be determined by a few general, pathological and pharmacodynamic notions, which are often imaginary, and are but too apt to lead astray, but it must be chosen in strict accordance with Hahnemann's rules.

2. Only one, or at the utmost two, globules of the proper remedy ought to be administered.

3. Await the effect patiently; in chronic diseases, seven, nine, twelve days; never interfere with an improvement, even though slight and just commencing, and progressing but slowly. If after the lapse of that period, no change occurs, then consider this as a proof that the remedy has been improperly selected, and that another ought to be selected. *Every hasty change of remedies inflicts incalculable injury.*

Repetitions are scarcely necessary, except in old diseases of the skin characterized by torpor. In acute cases, a new remedy may be given at the end of two or four hours, provided *no change whatever* takes place.—*Stapf.*

